# Assessment of image quality and impact of deep learning-based software in non-contrast head CT scans

**DOI:** 10.1038/s41598-024-62394-4

**Published:** 2024-05-23

**Authors:** Denise Bos, Aydin Demircioğlu, Julia Neuhoff, Johannes Haubold, Sebastian Zensen, Marcel K. Opitz, Marcel A. Drews, Yan Li, Hanna Styczen, Michael Forsting, Kai Nassenstein

**Affiliations:** 1 Institute of Diagnostic and Interventional Radiology and Neuroradiology, University Hospital Essen, University Duisburg-Essen, Hufelandstraße 55, 45147 Essen, Germany; 2 Faculty of Medicine, University Duisburg-Essen, Hufelandstraße 55, 45122 Essen, Germany

**Keywords:** Medical research, Software

## Abstract

In this retrospective study, we aimed to assess the objective and subjective image quality of different reconstruction techniques and a deep learning-based software on non-contrast head computed tomography (CT) images. In total, 152 adult head CT scans (77 female, 75 male; mean age 69.4 ± 18.3 years) obtained from three different CT scanners using different protocols between March and April 2021 were included. CT images were reconstructed using filtered-back projection (FBP), iterative reconstruction (IR), and post-processed using a deep learning-based algorithm (PS). Post-processing significantly reduced noise in FBP-reconstructed images (up to 15.4% reduction) depending on the protocol, leading to improvements in signal-to-noise ratio of up to 19.7%. However, when deep learning-based post-processing was applied to FBP images compared to IR alone, the differences were inconsistent and partly non-significant, which appeared to be protocol or site specific. Subjective assessments showed no significant overall improvement in image quality for all reconstructions and post-processing. Inter-rater reliability was low and preferences varied. Deep learning-based denoising software improved objective image quality compared to FBP in routine head CT. A significant difference compared to IR was observed for only one protocol. Subjective assessments did not indicate a significant clinical impact in terms of improved subjective image quality, likely due to the low noise levels in full-dose images.

## Introduction

Computed tomography (CT) scans of the head are an integral part of routine clinical practice and are essential for diagnosing conditions such as stroke, hemorrhage, seizures and tumors^1–3^. However, CT uses ionizing radiation, which is considered harmful because it is suspected of increasing the risk of cancer^4–7^. To mitigate this risk, radiation levels are reduced, resulting in a lower signal-to-noise ratio (SNR) and therefore more noise in the image. This can be particularly challenging in the brain, where noise can obscure details and potentially reduce the accuracy of the diagnosis.

Advanced image reconstruction techniques have been developed to reduce the impact of noise on CT images. These techniques can improve the overall image quality from the raw radiation data. One such technique is iterative reconstruction (IR), which has become widely available on newer scanners^8^. IR uses a mathematical algorithm to iteratively refine the reconstructed image, resulting in images that are less susceptible to noise than the standard filtered-back projection (FBP)^9^. In a systematic review published in 2015, the mean effective dose for contrast-enhanced CT of the chest was reduced by 50% with the use of IR compared to FBP^10^. Despite its advantages, IR has certain disadvantages. The algorithm requires more computing resources and is slower than FBP. In addition, IR can introduce artifacts into the reconstructed images, which can affect their diagnostic accuracy. The resulting images also tend to be smoother, which can obscure important details^8,11^. IR has become the standard of care for reconstructed CT images. However, in the continuous evolution of CT imaging from measured data to algorithmically improved data, FBP is the closest to the actual measured data.

Another way to reduce image noise, especially on older scanners where improved reconstruction algorithms are not available, is to use image-processing algorithms. These methods are applied after the CT scan has been acquired and attempt to reduce noise through statistical analysis without compromising diagnostic accuracy. Examples of such algorithms include relatively simple methods such as median filtering, and more complex models that are closely modeled on the specific statistics of the images. However, these methods can easily introduce artifacts if the processed images do not fit the assumed model. Recently, especially in the context of image processing, deep learning methods have been introduced to improve statistical denoising^12–17^. The advantage of these methods is that they do not require the specification of a noise model. They can infer it from given training data and could therefore be capable of denoising without introducing artifacts.

Deep learning-based image denoising (DLID) algorithms have only recently been introduced. It remains unclear whether they can fulfill the promise of improved image quality without compromising diagnostic accuracy and how large their impact is on full-dose CT images. Therefore, in this study, we aimed to assess the image quality processed by FBP, IR or a commercial DLID software. We compared the noise levels and clinical utility of these methods on a cohort of non-contrast brain CTs from three different sites and CT scanners.

## Results

**Figure 1:**
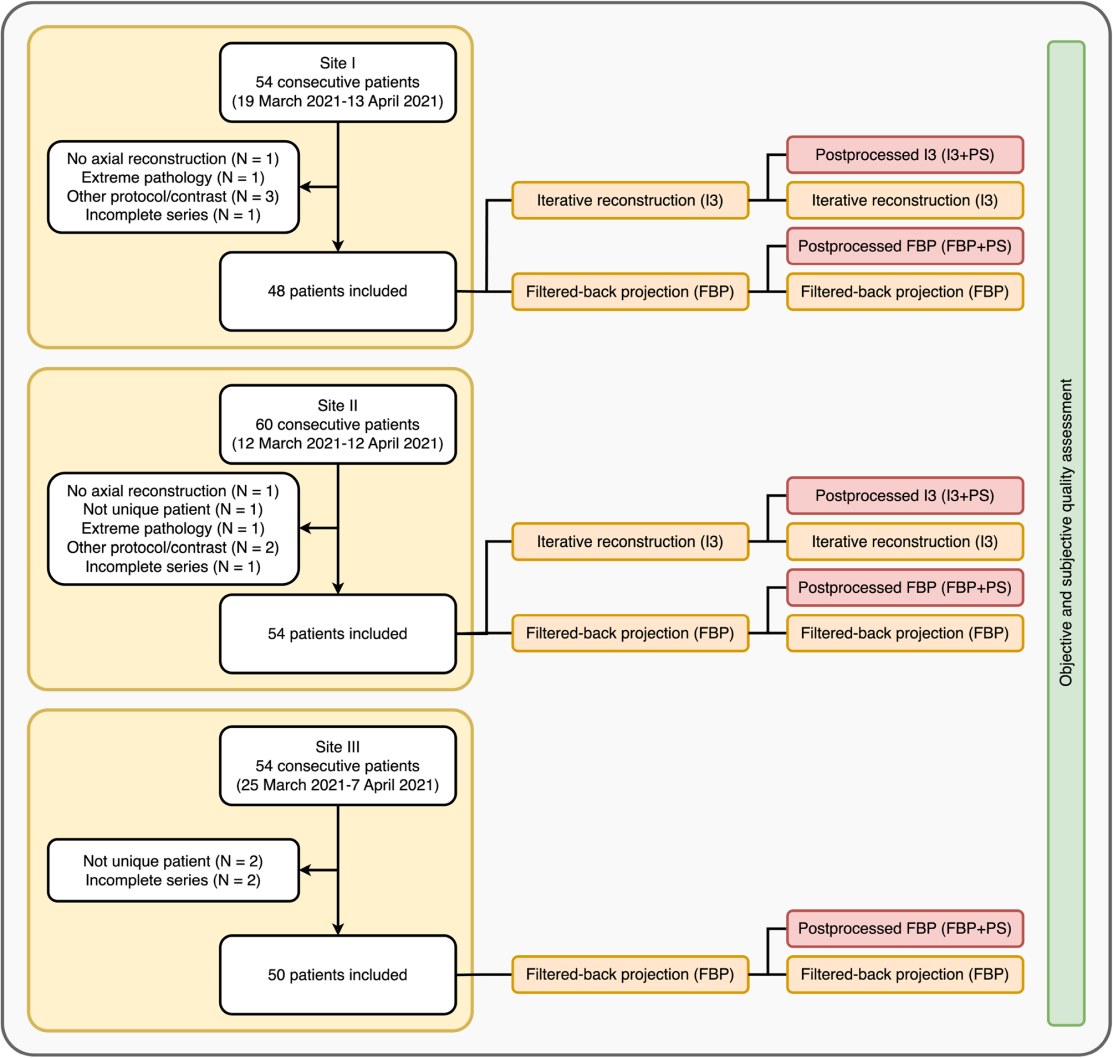
Patient flowchart and study design.

A total of 152 patients were included in this study (Fig. 1). The overall mean age of the patients was 69.4 ± 18.3 years, the distribution of females and males was approximately equal (77/75, 50.7%/49.3%) (Table 1). Radiation dose in terms of volumetric CT dose index (CTDI_vol_), dose-length product (DLP), and effective dose was significantly different between sites and hence protocols. Median CTDI_vol_ ranged from 30 to 48 mGy, median DLP from 498 to 847 mGy·cm, and median effective dose from 1.00 to 1.69 mSv (Table 1).

**Table 1:** Patient and scanner characteristics.

		Centre			*p*-Value (Site I vs. II, Site I vs. III, Site II vs. IIII)
		Site I	Site II	Site III	
Patient cohort	N	48	54	50	
	Male/Female (Percent)	20/28 (42%/58%)	28/26 (52%/48%)	27/23 (54%/46%)	0.41/0.31/0.98
	Median Age (IQR)	63 (50; 77)	82 (68; 88)	73 (59; 83)	** < 0.001/0.01/0.01**
Scan parameters	Reference mAs (A)/ Reference mAs (B)	300/300	273	–	–
	Median mAs (A) (IQR)/ Median mAs (B) (IQR)	213 (202; 224)/ 226 (215; 239)	214 (204; 221)	230 (230; 230)	–
	Median scan length in cm (IQR)	16.6 (15.8; 17.1)	16.6 (16.0; 17.5)	16.9 (15.8; 17.6)	0.59/0.30/0.60
Radiation dose	Median CTDI_vol_ (IQR) (mGy)	33 (31; 34)	30 (29; 31)	48 (48; 52)	** < 0.001/ < 0.001/ < 0.001**
	Median DLP (IQR) (mGy∙cm)	528 (501; 583)	498 (469; 532)	847 (798; 847)	** < 0.001/ < 0.001/0.001**
	Median Effective Dose (mSv) (IQR)*	1.06 (1.00; 1.17)	1.00 (0.94; 1.06)	1.69 (1.60; 1.69)	** < 0.001/ < 0.001/0.001**

*Effective Dose was calculated using the E/DLP coefficient (0.002 mSv/mGy·cm) from Shrimpton et al.^30^.

Significant values are in [bold].

### Objective image quality assessment

The absolute and relative differences in mean intensities across all regions of interest (ROIs) at all three sites were small and not significant, except for the comparison between deep-learning based, post-processed FBP images (FBP + PS) and iterative reconstructed images with strength three (I3) on the protocol/site II (Table 2). This appears to be due to a greater difference in mean intensities of the lateral ventricular anterior horn (LV), which was considerably smaller than the other areas (Table 6) and therefore showed greater variation (Table S1; Supplementary Information 1).

**Table 2:** Results of the objective image quality assessment.

Site	Method	Mean intensity difference over all ROIs except air	Mean noise difference over all ROIs except air	Mean SNR difference over all ROIs except air	Mean difference of CNR_infra_	Mean difference of CNR_supra_
Site I	FBP vs. FBP + PS	0.1 ± 0.6 (p = 0.935)	− 15.4 ± 7.4 (**p < 0.001**)	19.7 ± 11.2 (**p < 0.001**)	18.2 ± 7.9 (**p < 0.001**)	13.7 ± 6.5 (**p < 0.001**)
	FBP + PS vs. I3	0.2 ± 0.7 (p = 0.586)	1.0 ± 7.4 (p = 0.991)	0.1 ± 7.4 (p = 1.0)	− 1.7 ± 8.8 (p = 0.935)	0.9 ± 8.4 (p = 0.985)
	I3 + PS vs. I3	0.1 ± 0.5 (p = 0.991)	2.5 ± 5.0 (**p < 0.001**)	− 2.1 ± 4.0 (**p = 0.002**)	− 4.7 ± 6.9 (**p < 0.001**)	− 2.6 ± 4.1 (**p < 0.001**)
Site II	FBP vs. FBP + PS	0.2 ± 0.7 (p = 0.824)	− 8.5 ± 4.2 (**p < 0.001**)	10.1 ± 5.2 (**p < 0.001**)	13.4 ± 6.7 (**p < 0.001**)	4.3 ± 4.0 (**p < 0.001**)
	FBP + PS vs. I3	− 0.5 ± 0.8 (**p = 0.005**)	− 11.1 ± 4.6 (**p < 0.001**)	13.0 ± 5.8 (**p < 0.001**)	2.9 ± 10.0 (p = 0.167)	8.8 ± 5.3 (**p < 0.001**)
	I3 + PS vs. I3	− 0.1 ± 0.7 (p = 0.969)	− 0.4 ± 1.5 (p = 0.878)	0.5 ± 1.9 (p = 0.935)	− 0.9 ± 5.4 (p = 0.957)	− 0.6 ± 2.5 (p = 0.903)
Site III	FBP vs. FBP + PS	0.3 ± 0.8 (p = 0.388)	− 10.5 ± 7.6 (**p < 0.001**)	13.4 ± 10.1 (**p < 0.001**)	13.4 ± 8.7 (**p < 0.001**)	8.2 ± 7.9 (**p < 0.001**)

p-values were computed using a Wilcoxon test. Significant p-values are marked in boldface.

Regarding the noise, measured by the standard deviation, significant differences could be observed in all ROIs (Table 2). Especially in FBP + PS vs. FBP, the decreases in mean noise over all ROIs (except air) were significant: − 15.4 ± 7.4 (site I), − 8.5 ± 4.2 (site II), − 10.5 ± 7.6 (site III) (all *p* < 0.001) (Table 2). However, when comparing post-processed I3 images (I3 + PS) vs. I3 and FBP + PS vs. I3, non-significant decreases or even significant increases could be observed (Table 2), which did not seem to depend on the ROI but on the protocol/site (Table S4).

A similar pattern was observed for the signal-to-noise ratio (SNR); a strong and significant increase was observed when post-processing was applied to the FBP images, but non-significant increases or even decreases were observed for the other comparisons (Table 2). Again, these differences were not strongly dependent on the ROI, but on the protocol/site (Table S6).

For both contrasts, contrast-to-noise ratios infra- and supratentorial (CNR_infra_ and CNR_supra_), a clear and significant increase was observed when comparing FBP vs. FBP + PS (Table 2). When comparing FBP + PS vs. I3, some increase was observed on site II, but almost none on site I. However, when comparing I3 + PS vs. I3, a partially non-significant decrease was observed in all cases.

### Subjective image quality assessment

Concordance between raters for subjective image quality ranking was rather low (W < 0.33 in all cases) and almost always insignificant, indicating no agreement between raters (Table 3). The disagreement was very pronounced when comparing FBP + PS vs. I3 in site II, where one rater preferred I3 in all cases, while another almost always preferred FBP + PS. This disagreement was also present at the other sites, although in a weaker form, and was also observed when comparing FBP and FBP + PS. However, a slight agreement (*p* = 0.02) was observed when comparing FBP to FBP + PS at site I, where three raters preferred FBP + PS to FBP. On the other hand, there was no clear agreement when comparing I3 + PS to I3 (rank difference < 0.27 for all raters). Pairwise Wilcoxon signed-rank tests showed no significant difference between FBP and FBP + PS (*p* = 0.08), or between FBP + PS and I3, or I3 and I3 + PS (both *p* > 0.50), indicating that no clear subjective preference for either of the methods was observed.

**Table 3:** Results of the difference in ranking for the subjective image quality assessment.

Site	Method	Rater 1	Rater 2	Rater 3	Rater 4	W	*p*
Site I	FBP vs. FBP + PS	− 0.58	− 0.85	0.31	− 0.42	0.33	**0.02**
	FBP + PS vs. I3	− 0.06	− 0.79	0.15	− 0.25	0.29	0.11
	I3 + PS vs. I3	0.27	− 0.06	0.12	0	0.29	0.12
Site II	FBP vs. FBP + PS	− 0.09	− 0.62	0.2	0.05	0.26	0.4
	FBP + PS vs. I3	0.94	− 1	0.8	− 0.04	0.13	1
	I3 + PS vs. I3	0.17	− 0.02	0.07	0.04	0.25	0.47
Site III	FBP vs. FBP + PS	− 0.31	− 0.73	0.47	0.04	0.23	0.67

Positive values for the raters indicate that the images of the first method were rated higher. W indicates the concordance of the ratings computed by Kendall’s W. A p-value less than 0.05 indicates agreement and is shown in bold.

Overall subjective image quality was rated most differently when comparing FBP + PS vs. I3, where the concordance was lowest (W = 0.21) (Table 4). Since two raters did not see a difference in overall image quality in all comparisons, the concordance depended mainly on the ratings of the other two raters; concordance was observed in almost all comparisons. Rater 1 rated FBP + PS as much better in terms of overall image quality than I3 at site II, but rated them similarly at site I. In comparison, rater 4 rated I3 better in overall image quality at site II and also at site I, although less clearly. Pairwise Wilcoxon signed-rank tests showed no significant difference between FBP and FBP + PS, FBP + PS and I3, or I3 and I3 + PS (all *p* > 0.50), indicating that no method was rated to be better in terms of subjective overall image quality than the other.

**Table 4:** Results of the difference in overall image quality for the subjective image quality assessment.

Site	Method	Rater 1	Rater 2	Rater 3	Rater 4	W	*p*
Site I	FBP vs. FBP + PS	-0.33	0	− 0.02	− 0.54	0.44	** < 0.001**
	FBP + PS vs. I3	0.04	0	0	− 0.46	0.34	**0.01**
	I3 + PS vs. I3	0.08	0	0	− 0.23	0.41	** < 0.001**
Site II	FBP vs. FBP + PS	− 0.04	0	0	− 0.11	0.30	0.07
	FBP + PS vs. I3	0.74	0	0	− 0.81	0.21	0.89
	I3 + PS vs. I3	0.02	0	0	0.02	0.36	** < 0.001**
Site III	FBP vs. FBP + PS	− 0.20	0	0	− 0.22	0.43	** < 0.001**

Positive values for the raters indicate that the images of the first method were rated higher. W indicates the concordance of the ratings calculated by Kendall’s W. A *p*-value less than 0.05 indicates agreement and is shown in bold.

## Discussion

In this study, we compared different reconstruction algorithms with a DLID post-processing software. Our results indicated that using post-processing can reduce the overall noise level in filtered-back projected non-contrast head CTs to a level that is comparable with iterative reconstruction.

As expected, neither a different reconstruction algorithm nor applying post-processing does change the overall mean of the intensities in each ROI significantly, as nearly all differences are below 3%. The only exception is the intensity in the lateral ventricle anterior horn (LV) where differences of up to 5.5% were observed; however, since this ROI was the smallest with just 30 mm^2^, this might be a statistical effect.

When considering the noise, measured by the standard deviation, a large reduction could be observed when applying post-processing to the FBP reconstructed images (reduction up to 15.4%, ignoring air). However, these reductions seemed to depend on the scanner and protocol. Yet, comparing the post-processed FBP images to the I3 images, a further reduction could be seen in only one site. This indicates that the noise level of the I3 images is already very low, and might express the over-smoothed impression in these images^8,11^. Also, applying post-processing to the iterated images did not clearly result in a further noise reduction, in other words, the post-processing did not seem to result in additional smoothing. It remains unclear why there is an inconsistent improvement in the post-processed FBP images compared to I3. This may be due to different CT protocols with significantly different radiation doses, or to the fact that the noise level in the CT images examined was generally very low. In further studies, different protocols on the same scanners with different dose requirements need to be examined in this comparison to be able to say definitively whether the post-processed FBP images are better than the I3 images. The differences may be clearer in low-dose CT examinations, as Drews et al. have previously shown in low-dose abdominal CT, where post-processed FBP images (using the same software) were superior to I3 images in terms of noise and SNR^18^.

Similar observations were true for the signal-to-noise ratio as well: In nearly all ROIs, applying post-processing to the FBP reconstructed images resulted in an increase of the signal-to-noise ratio (up to 17.5%, ignoring air). When comparing the post-processed FBP images to I3, however, a further increase of the signal-to-noise ratio was seen. Again, while this seems to indicate that the I3 images are superior, the smoothing might already be at a level that impairs diagnosis of small pathologies. Furthermore, applying post-processing to the iterated reconstructions did not yield a further increase, i.e., it did not change the overall impression.

Surprisingly, the subjective ratings were not as coherent. The raters seemed to have different preferences. Some preferred the noisier CT images of FBP, some the less noisy, post-processing FBP or the smoother, iterative reconstructed images I3. In addition, the ranking was also not consistent. Concordance between raters was mostly insignificant, meaning that we could not observe a higher correlation between the raters across all sites and methods. Only the comparison for FBP and the post-processed FBP + PS was statistically significant (*p* = 0.02). Regarding the subjective overall image quality, again, we could not see any difference, although the difference between FBP and post-processing FBP barely missed significance (*p* = 0.08). In addition, we could not see a relation between the ratings and the rater’s experience.

These results indicate two implications for the clinical routine: First, post-processing based on deep learning is able to reduce the noise, when it is measured objectively in terms of standard deviation. Second, reduced noise levels do not transfer directly to an improved subjective overall image quality of the images. This might be due to the fact that overly smoothed images do have lower noise levels, but at the same time obstructs the diagnosis by blurring. However, this seemed to depend strongly on the rater and on the protocol/site, and not so much on the experience of the reader. Differences in the rating of the reconstructed and post-processed images might depend also on the anatomic region and that radiologist are used to and prefer a specific noise level in their CT images. In addition, due to the low noise level, the noise reduction was not as high as in low-dose or ultra-low-dose CT images, so subjective differences may not be visible to the human eye, which could result in low inter-rater reliability and non-significant results in subjective image quality testing^13,14,19^.

A similar study has been performed recently, comparing the post-processing for low-dose abdominal CTs by Steuwe et al.^13^. In soft tissue, the DLID algorithm reduced image noise by up to 42% compared to FBP and 27% compared to IR. This resulted in the algorithm achieving the highest SNR and CNR. Subjective image quality was equal between IR and the algorithm, both surpassing the image quality of FBP.

In their study of low-dose whole-body CT scans for melanoma patients, Brendlin et al. found that the DLID software consistently maintained good to excellent subjective image quality^14^. Objective analysis showed significant noise reduction in all post-processed images compared to standard reconstruction, regardless of radiation dose or scanner type. Most importantly, the software enabled diagnostic images with radiation doses as low as 30% of the initial dose, demonstrating its potential to significantly reduce patient radiation exposure, particularly in repeat whole-body staging exams. In another study, Brendlin et al. showed similar results on pediatric ultra-low-dose chest CT^19^. The software substantially reduced image noise compared to conventional methods, resulting in higher subjective image quality ratings. It also significantly accelerated the time to diagnosis without compromising diagnostic confidence. Lyoo et al. combined an ultra-low dose CT protocol with deep learning reconstruction and were able to improve image quality to a level comparable to the full-dose CT protocol without compromising diagnostic accuracy for craniosynostosis in children^20^.

Hong et al. combined DLID with IR and were able to further reduce noise and improve subjective and objective image quality of coronary CT angiographies^21^. Kim et al. also compared brain CT images reconstructed using two methods: deep learning-based image reconstruction and adaptive statistical iterative reconstruction-Veo (ASIR-V)^12^. Medium and high-strength deep learning-based reconstructed images had the best subjective image quality scores among the reconstruction datasets; it reduced image noise and artifacts while improving image quality compared to ASIR-V. However, we did not see an overall statistically significant improvement compared to IR in our study.

The focus of our study was the investigation of the image quality of DLID images compared to FBP and IR, however, DLID is also relevant in radiomics. For example, Zhong et al. demonstrate their deep learning image reconstruction algorithm on raw CT data leads to more robust radiomic features^22^. ASIR-V images exhibited greater consistency with other traditional IR methods when compared to deep learning-based reconstructed images. While the deep learning-based reconstruction algorithm might introduce changes to radiomic features when contrasted with IR algorithms, their analysis identified nine radiomics features that remained consistent and reproducible.

Besides the reviewed and commercially available DLID software, there are other denoising software such as ClariCT.AI (ClariPi, Seoul, South Korea)^21,23,24^, AiCE (advanced intelligent Clear-IQ Engine, Canon, Tokyo, Japan)^25–27^, or TrueFidelity™ (GE Healthcare, Chicago, USA)^28,29^, or non-purchasable, self-developed DLID. The number of algorithms will certainly increase rapidly. The quality of the data used for model training is critical to the performance of the DLID algorithms. With photon counting CT scanners, higher quality training data will be available in the near future. At the same time, spectral data would benefit from the computational power of deep learning reconstruction techniques^30^. It will be interesting to compare or combine the different algorithms.

Our study has a few limitations. First, we measured noise only in terms of standard deviation in specific, manually signed ROIs. Other options would have been automatic noise quantification methods, such as measurements from automated body and organ analysis. Aspects of image quality, such as resolution or blurring effects, are not considered in the objective image quality assessment performed, but play a role in the subjective image quality assessment. No comparison was made with measurements on phantoms. Then, our deep learning-based noise reduction is from a commercial vendor, and we have no insight into the algorithms and their inner workings. In addition, the software may have improved in the meantime, and other algorithms and commercial products have appeared on the market. In further studies, it would be interesting to compare different products and protocols on various CT scanners from different manufacturers. Another limitation is that subjective ratings are prone to potential rater bias. We minimised this by presenting the CT images in a blinded fashion. Other options would have been additional external raters or multiple raters with different experience, although we had four, which is usually more than in other published studies. Also, different aspects of image quality such as artefacts or resolution were not taken into account in the ratings, only overall subjective image quality measured on a 5-point Likert scale. The main strength of the examined software is also the denoising of low-dose and ultra-low-dose CT images with high levels of image noise^13,14,19^. For these use cases, the software also offered potential benefits in terms of subjective image quality. Nevertheless, we have tested the subjective image quality only on non-contrast head CT images with full-dose and low noise levels.

In conclusion, we investigated the objective and subjective image quality of non-contrast head CT images on three different CT scanners and protocols reconstructed with filtered-back projection, iterative reconstruction, and post-processed with a deep learning-based reconstruction algorithm. Objective image quality was improved by the deep learning-based reconstruction technique compared to filtered-back projection, which is especially helpful for older CT scanners that do not have iterative reconstruction. Compared to iterative reconstruction techniques, there was no overall statistically significant difference using the deep learning denoising software, which may be scanner/protocol specific. Subjective assessments did not indicate a significant clinical impact in terms of improved subjective image quality, likely due to low noise levels in full-dose images or radiologist preference.

## Materials and methods

This retrospective study was approved by the local ethics committee (Faculty of Medicine, University of Duisburg-Essen; number 21-9996-BO). The requirement to obtain informed consent was waived. All procedures performed in the studies involving human participants were in accordance with the ethical standards of the institutional and/or national research committee and with the Helsinki Declaration of 1964 and its later amendments or comparable ethical standards.

### Patient cohort

Patient data were collected at three different sites of one radiological institution (site I: University Hospital Essen, Essen, Germany; site II: Elisabeth Hospital, Essen, Germany; site III: St. Marien-Hospital, Mülheim an der Ruhr, Germany) between March and April 2021. Collection was performed consecutively, until around 50 patients were included. Patients were considered eligible if they underwent a routine head CT scan without contrast. Examinations were identified using the DICOM header-based dose monitoring software Radimetrics (Bayer AG, Leverkusen, Germany) and the local picture archiving and communications system (PACS). Studies were excluded if brain pathology significantly affected the objective assessment of image quality (e.g., severe cerebral edema or hemorrhage). Incomplete CT scans or repeated scans of the same patient during the study period were also excluded. CT scans that were incompletely post-processed by the DLID software or where no post-processed images were generated on the axial reconstructed CT images were also excluded.

### CT protocols and scanner

Head CTs were performed without contrast material on three different CT scanners from one manufacturer (Siemens Healthineers, Erlangen, Germany): a dual-source 192-slice SOMATOM Force (site I), a dual-source 128-slice SOMATOM Definition Flash (site II), and a single-source 6-slice SOMATOM Emotion 6 (site III) (Table 5). Scans on the SOMATOM Force were performed in dual-energy mode, all others in single-energy mode. Automatic tube current modulation (CARE Dose 4D, Siemens Healthineers, Erlangen, Germany) was performed on the SOMATOM Force and SOMATOM Definition Flash.

**Table 5:** Overview of CT scanner and scanning parameters.

		Centre		
		Site I	Site II	Site III
CT scanner	Manufacturer	Siemens	Siemens	Siemens
	Model	SOMATOM Force	SOMATOM Definition Flash	SOMATOM Emotion 6
	Slices	192	128	6
	Sources	dual	dual	single
	Mode	spiral	spiral	sequential
Technical parameters	Reconstructed slice thickness (mm)	5	5	4
	Single collimation width (mm)	0.6	0.6	2
	Total collimation width (mm)	19.2	38.4	12
	Pitch factor	0.7	0.6	–
	Mode	dual energy	single energy	single energy
	kVp (A)/ kVp (B)	90/150	120	130
	Reference mAs (A)/ reference mAs (B)	300/300	273	–

### Radiation dose assessment

Radiation doses for the dose metrics CTDI_vol_ and DLP were extracted from the dose sheet of each CT scan. The effective dose (E) was calculated using the E/DLP coefficient (0.002 mSv/mGy·cm) from Shrimpton et al.^31^.

### Reconstruction and post-processing

Each CT scan was reconstructed using either FBP only or both FBP and iterative reconstruction (IR) with strength three (I3) out of five, depending on availability. Reconstruction was performed using the scanner manufacturer’s software, which was ADMIRE (advanced modeled iterative reconstruction, site I) and SAFIRE (sinogram affirmed iterative reconstruction, site II) from Siemens Healthineers (Erlangen, Germany). The CT scanner on site III did not have an iterative reconstruction.

**Figure 2:**
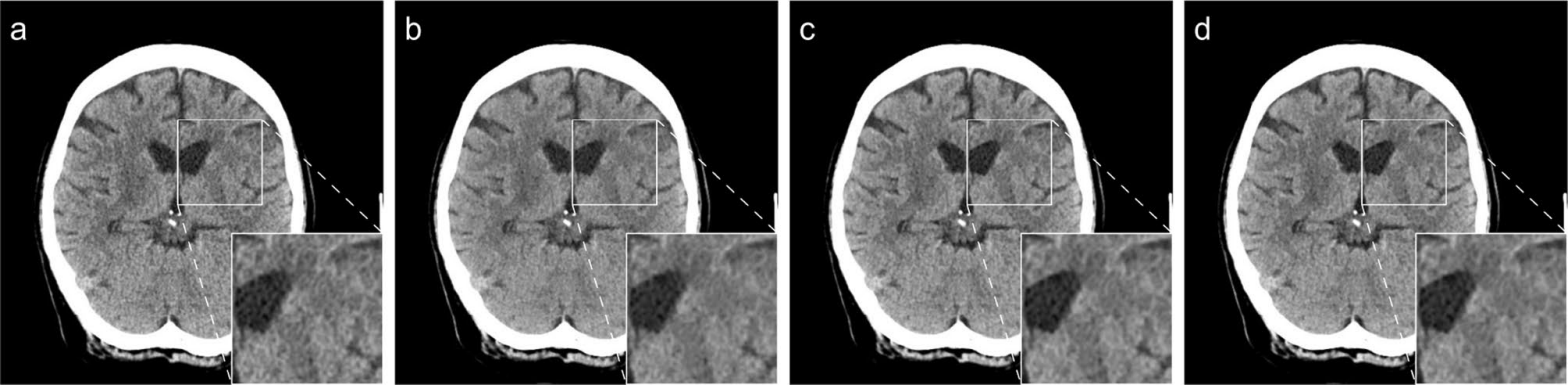
Example of four differently reconstructed and post-processed images from the same CT scan (Siemens SOMATOM Force, site I). In each figure, a section of the images is zoomed and shown in the lower right corner. (**a)** Image reconstructed with filtered-back projection (FBP). **(b)** Image reconstructed with filtered-back projection and post-processed (FBP + PS). (**c)** Image reconstructed with iterative reconstruction (I3). (**d)** Image reconstructed with iterative reconstruction and post-processed (I3 + PS).

The reconstructed scans were then post-processed using the deep learning-based reconstruction algorithm (PS), resulting in two additional CT images, the post-processed FBP scan (FBP + PS) and the I3 scan (I3 + PS). Post-processing was performed using the commercial software PixelShine v1.3 (AlgoMedica Inc, Sunnyvale, CA, USA), which takes only a few seconds after manual sending to the software. The default parameters for brain CT were left unchanged. The following head reconstruction parameters were used: streak artifact reduction of R1 (weakest), sharpening level of P2, target noise level of 3.5 (based on Hounsfield unit (HU)), and maximum processing strength of H9. The software estimates the noise in the original CT images and then adapts the amount of processing required for the target noise level. Figure 2 shows an example of the different image reconstructions and post-processing.

### Objective image quality assessment

**Figure 3:**
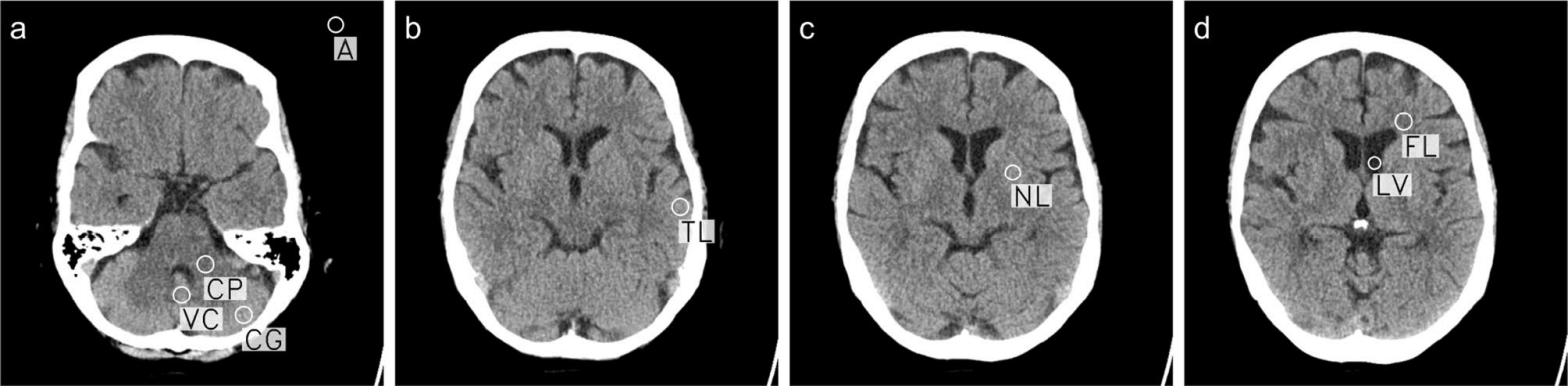
Example of an annotation of a patient. (**a)** Slice containing the region of white matter cerebellar peduncle (CP), the vermis cerebelli (VC), the cerebellum gray matter (CG), and air (A). **(b)** Slice containing the temporal lobe gray matter (TL). (**c)** Slice containing the nucleus lentiformis (NL). (**d)** Slice containing the frontal lobe white matter (FL) and the lateral ventricle anterior horn (LV).

Noise was assessed by measuring HU values in several ROIs important for clinical diagnosis and image quality according to Wu et al.^32^ (Fig. 3). Each region was labeled by a radiologist and a resident in consensus using ROIs with a fixed size between 30 and 65 mm^2^, depending on the tissue type and location (Table 6). ROIs were annotated in the FBP-reconstructed scan and then automatically copied to all other scans, so that for each scan all ROIs were annotated in the same position (Fig. 3). A custom tool written in Python was used (Figure S1; Supplementary Information 1).

**Table 6:** Overview of annotated regions of interest (ROI) and their median size.

Region	Median area (IQR) [mm^2^]
Nucleus lentiformis (NL)	53.3 (53.0; 53.3)
Frontal lobe white matter (FL)	64.1 (63.8; 64.4)
Temporal lobe gray matter (TL)	65.9 (65.6; 66.2)
Lateral ventricle anterior horn (LV)	30.2 (30.2; 30.3)
Cerebellum gray matter (CG)	60.4 (60.2; 60.4)
White matter cerebellar peduncle (CP)	59.4 (59.2; 59.5)
Vermis cerebelli (VC)	60.4 (60.2; 60.4)
Air (A)	45.7 (45.7; 45.9)

The size of each ROI was similar in each scan, however due to different pixel spacing it was not equal.

To assess the amount of noise, the mean intensity signal and its standard deviation were measured in each of the ROIs. The standard deviation can be considered as a surrogate for the noise level. In addition, the SNR was calculated by dividing the mean by the standard deviation. In addition, two contrast-to-noise ratios were calculated, which measure the contrast in the image by differences in the intensity of related tissues: CNR_supra_, between the frontal lobe white matter (FL) and the temporal lobe gray matter (TL), and CNR_infra_, between the cerebellum gray matter (CG) and the white matter cerebellar peduncle (CP):

$$CNR_{supra} = \frac{{ \mu_{TL} - \mu_{FL} }}{{\sqrt {\sigma_{TL}^{2} + \sigma_{FL}^{2} } }}\;and\;CNR_{infra} = \frac{{ \mu_{CG} - \mu_{CP} }}{{\sqrt {\sigma_{CG}^{2} + \sigma_{CP}^{2} } }}$$

where *µ* denotes the mean intensity and $\sigma$ denotes the standard variation in the corresponding region (according to Wu et al.^32^).

### Subjective image quality assessment

To determine whether the reconstructed or post-processed images were suitable for clinical routine, subjective image quality assessments were performed by four raters, two of whom were experienced neuroradiologists (three raters from site I, one from site II/III). In a custom-tailored, web-based tool, images at the height of the lateral ventricle anterior horn (LV) were displayed in a blinded fashion without any annotation and randomly (Figure S2; Supplementary Information 1). The rater was then asked to rank the images (1 for the better image, 2 for the worse image). Equal ranks were allowed (rating 1 two times). In addition, the overall subjective image quality of each image was rated on a 5-point Likert scale (with options “excellent”, “completely acceptable”, “mostly acceptable”, “suboptimal”, “unacceptable”). Only overall image quality was rated, and specific aspects of image quality such as artefacts were not considered.

In order to measure whether applying DLID post-processing to the images is helpful, three tasks were considered: First, FBP images were compared with post-processed FBP images (FBP vs. FBP + PS). This task was included to determine if applying denoising could improve image quality on older scanners where I3 is not available. Then, I3 images were compared to post-processed I3 images (I3 vs. I3 + PS). This task was performed to determine if applying the denoising software to IR images provided any additional benefit. Readers were also asked to rate the quality of post-processed FBP images compared to I3 (FBP + PS vs. I3). This task was included to determine if the application of the denoising software could achieve the same image quality as modern scanners where I3 is available. In all tasks, all available images were evaluated, regardless of the site where the image was acquired.

### Statistics

Descriptive statistics were reported as mean and standard deviation or median and interquartile range (IQR). Wilcoxon or χ^2^ tests were used to compare demographics. Concordance between subjective ratings were compared using Kendall’s coefficient of concordance W. Preference for the different methods was measured using a Wilcoxon signed-rank test. A *p*-value < 0.05 was considered statistically significant. Statistical analysis was performed using R v4.2.0.

## Ethics approval

This study was performed in line with the principles of the Declaration of Helsinki. Approval was granted by the Ethics Committee of the Medical Faculty of the University of Duisburg-Essen (21–9996-BO).

## Informed consent

Informed consent was waived by the local Ethics Committee of the Medical Faculty of the University of Duisburg-Essen in view of the retrospective nature of the study and all the procedures being performed were part of the routine care.

### Supplementary Information

Supplementary Information.

## Data Availability

The data analyzed in this study is not publicly available due to privacy and security concerns. The data may be shared with a third party upon execution of data sharing agreement for reasonable requests, such requests should be addressed to D.B. (e-mail: denise.bos@uk-essen.de).

## Code availability

The code may be made available from the authors upon reasonable request subject to permission and approval from the corresponding organizations and institutional review boards.

## References

[1] Smits M (2007). Minor head injury: Guidelines for the use of CT—a multicenter validation study. Radiology.

[2] American College of Radiology. ACR appropriateness criteria. (2012) https://www.acr.org/Clinical-Resources/ACR-Appropriateness-Criteria, Accessed 09-13-2023.

[3] Powers WJ (2019). Guidelines for the early management of patients with acute ischemic stroke: 2019 update to the 2018 guidelines for the early management of acute ischemic stroke: A guideline for healthcare professionals from the American heart association/American stroke association. Stroke.

[4] International Agency for Research on Cancer. (2012). Radiation—IARC monographs on the evaluation of carcinogenic risks to humans. International Agency for Research on Cancer, Lyon, France. PMC7681469 1683674

[5] National Research Council (2006). Health Risks from Exposure to Low Levels of Ionizing Radiation: BEIR VII Phase 2.

[6] Pearce MS (2012). Radiation exposure from CT scans in childhood and subsequent risk of leukaemia and brain tumours: A retrospective cohort study. Lancet.

[7] Cardis E (2005). Risk of cancer after low doses of ionising radiation: Retrospective cohort study in 15 countries. Bmj.

[8] Geyer LL (2015). State of the art: Iterative CT reconstruction techniques. Radiology.

[9] Cho H-H, Lee SM, You SK (2020). Pediatric head computed tomography with advanced modeled iterative reconstruction: Focus on image quality and reduction of radiation dose. Pediatr. Radiol..

[10] den Harder AM (2015). Achievable dose reduction using iterative reconstruction for chest computed tomography: A systematic review. Eur. J. Radiol..

[11] Hardie AD, Nelson RM, Egbert R, Rieter WJ, Tipnis SV (2015). What is the preferred strength setting of the sinogram-affirmed iterative reconstruction algorithm in abdominal CT imaging?. Radiol. Phys. Technol..

[12] Kim I, Kang H, Yoon HJ, Chung BM, Shin N-Y (2021). Deep learning–based image reconstruction for brain CT: Improved image quality compared with adaptive statistical iterative reconstruction-Veo (ASIR-V). Neuroradiology.

[13] Steuwe A (2021). Influence of a novel deep-learning based reconstruction software on the objective and subjective image quality in low-dose abdominal computed tomography. Br. J. Radiol..

[14] Brendlin AS (2022). Ai denoising significantly improves image quality in whole-body low-dose computed tomography staging. Diagnostics.

[15] Arndt C (2021). Deep Learning CT Image Reconstruction in Clinical Practice. RöFo.

[16] Greffier J (2020). Image quality and dose reduction opportunity of deep learning image reconstruction algorithm for CT: A phantom study. Eur. Radiol..

[17] Alagic Z (2022). Deep learning versus iterative image reconstruction algorithm for head CT in trauma. Emerg. Radiol..

[18] Drews MA (2024). Impact of AI-based post-processing on image quality of non-contrast computed tomography of the chest and abdomen. Diagnostics.

[19] Brendlin AS (2022). AI denoising improves image quality and radiological workflows in pediatric ultra-low-dose thorax computed tomography scans. Tomography.

[20] Lyoo Y (2023). Ultra-low-dose computed tomography with deep learning reconstruction for craniosynostosis at radiation doses comparable to skull radiographs: A pilot study. Pediatr. Radiol..

[21] Hong JH, Park E-A, Lee W, Ahn C, Kim J-H (2020). Incremental image noise reduction in coronary CT angiography using a deep learning-based technique with iterative reconstruction. Korean J. Radiol..

[22] Zhong J (2023). Deep learning image reconstruction algorithm reduces image noise while alters radiomics features in dual-energy CT in comparison with conventional iterative reconstruction algorithms: A phantom study. Eur. Radiol..

[23] Nam JG (2021). Image quality of ultralow-dose chest CT using deep learning techniques: Potential superiority of vendor-agnostic post-processing over vendor-specific techniques. Eur. Radiol..

[24] Yeoh H (2021). Deep learning algorithm for simultaneous noise reduction and edge sharpening in low-dose CT images: A pilot study using lumbar spine CT. Korean J. Radiol..

[25] Noda Y (2021). Deep learning image reconstruction for pancreatic low-dose computed tomography: Comparison with hybrid iterative reconstruction. Abdom. Radiol..

[26] Singh R (2020). Image quality and lesion detection on deep learning reconstruction and iterative reconstruction of submillisievert chest and abdominal CT. Am. J. Roentgenol..

[27] Tanabe N (2022). Deep learning-based reconstruction of chest ultra-high-resolution computed tomography and quantitative evaluations of smaller airways. Respir. Investig..

[28] Kim JH (2021). Validation of deep-learning image reconstruction for low-dose chest computed tomography scan: Emphasis on image quality and noise. Korean J. Radiol..

[29] Park C (2021). CT iterative vs deep learning reconstruction: Comparison of noise and sharpness. Eur. Radiol..

[30] Koetzier LR (2023). Deep learning image reconstruction for CT: Technical principles and clinical prospects. Radiology.

[31] Shrimpton PC, Jansen JT, Harrison JD (2016). Updated estimates of typical effective doses for common CT examinations in the UK following the 2011 national review. Br. J. Radiol..

[32] Wu T-H (2013). How far can the radiation dose be lowered in head CT with iterative reconstruction? Analysis of imaging quality and diagnostic accuracy. Eur. Radiol..

