# Supplementary material for: Assessment of image quality and impact of deep learning-based software in non-contrast head CT scans
Source: Sci Rep. 2024 May 23;14:11810. doi: 10.1038/s41598-024-62394-4 (PMC11116440; doi:10.1038/s41598-024-62394-4)
Supplement: Supplementary file 1 — Supplementary Information. [file 41598_2024_62394_MOESM1_ESM.docx]

## Assessment of Image Quality and Impact of Deep Learning-Based Software in Non-Contrast Head CT Scans

Denise Bos, MD^1*^, Aydin Demircioğlu, PhD^1^, Julia Neuhoff, MD^2^, Johannes Haubold, MD^1^,
Sebastian Zensen, MD^1^, Marcel K. Opitz, MD^1^, Marcel A. Drews, MD^1^, Yan Li, MD^1^,
Hanna Styczen, MD^1^, Michael Forsting, MD^1^, Kai Nassenstein, MD^1^

**Affiliations**^1^ Institute of Diagnostic and Interventional Radiology and Neuroradiology, University Hospital Essen, University Duisburg-Essen, Hufelandstraße 55, 45147 Essen, Germany.

^2^ Faculty of Medicine, University Duisburg-Essen, Hufelandstraße 55, 45122 Essen

## Electronic supplemental material

##### Annotation tool


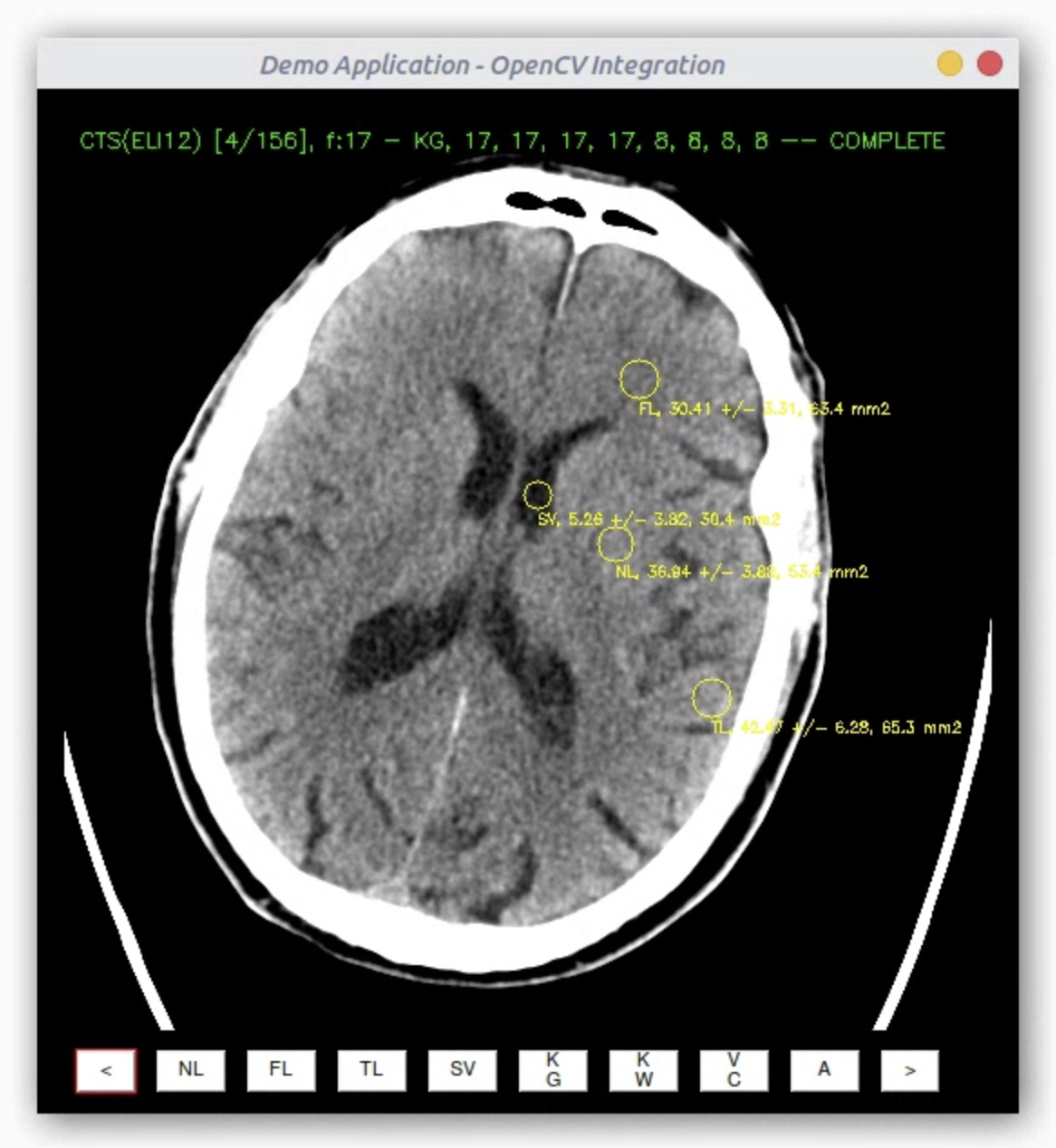


**Figure S1.** Screenshot of the custom-tailored Python tool for annotation of the ROIs.

##### Web-based rating tool

**
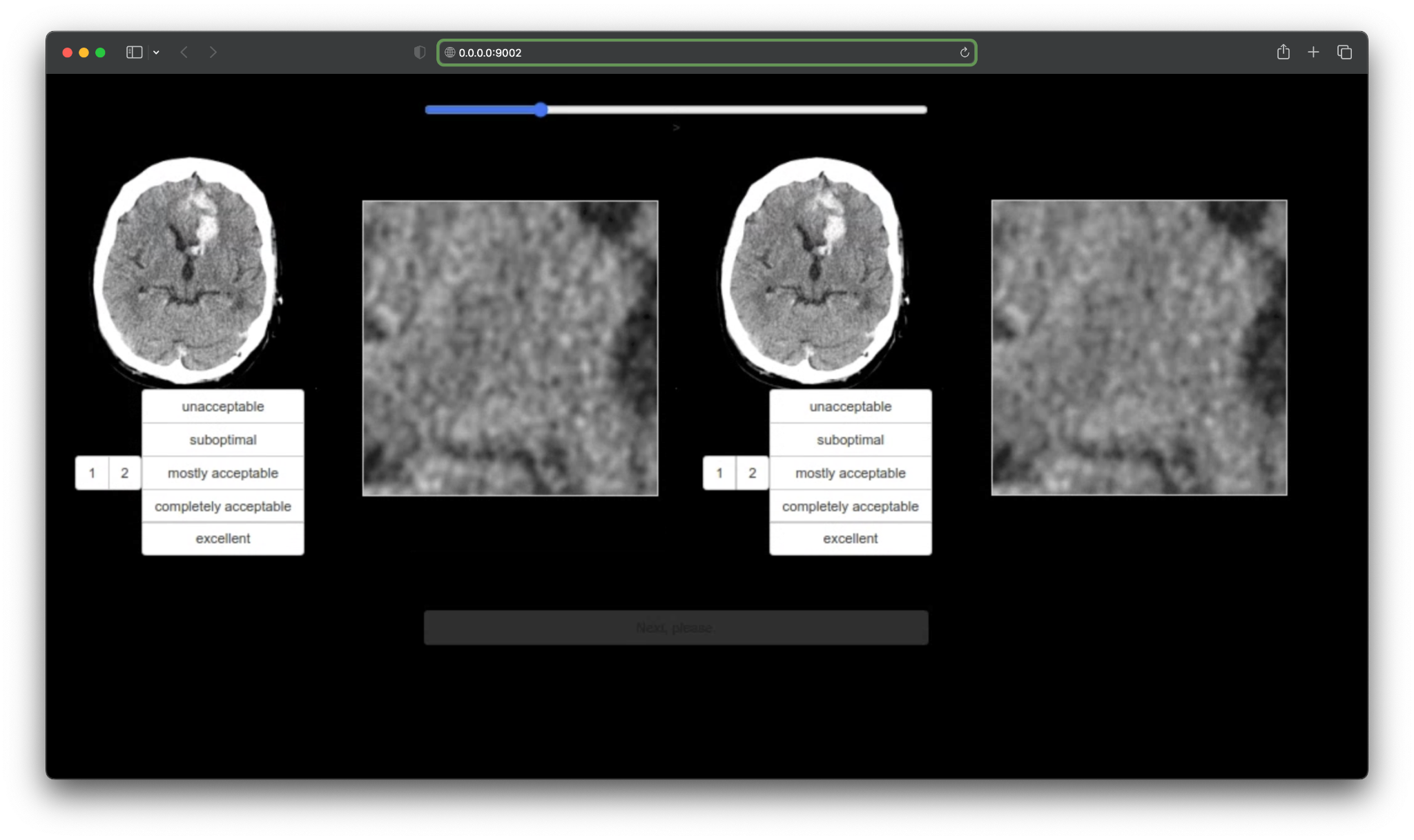
Figure S2.** Screenshot of the custom-tailored web-based rating tool for rating of the image quality. The zoomed areas interactively showed the area below the mouse pointer. The zoom level could be adjusted by the top slider. The button to continue to the next image was activated only after the annotation was completed.

#####

#####

##### Mean intensities

**Table S1.** Mean HU values and standard deviation for each ROI.

| **Site** | **Method** | **A** | **FL** | **CG** | **CP** | **NL** | **LV** | **TL** | **VC** |
| --- | --- | --- | --- | --- | --- | --- | --- | --- | --- |
| **I** | **FBP** | -1001.6±1.0 | 30.9±2.8 | 43.7±2.5 | 31.4±1.9 | 40.0±1.5 | 9.3±2.6 | 43.0±2.7 | 41.3±1.6 |
|  | **FBP+PS** | -1000.5±0.9 | 30.9±2.8 | 43.7±2.5 | 31.3±1.9 | 39.8±1.4 | 9.4±2.6 | 43.1±2.7 | 41.1±1.6 |
|  | **I3** | -1001.6±1.0 | 31.0±2.9 | 44.0±2.5 | 31.5±1.9 | 40.2±1.5 | 9.1±2.5 | 43.4±2.7 | 41.8±1.6 |
|  | **I3+PS** | -1000.6±0.9 | 31.0±2.9 | 44.1±2.5 | 31.4±1.9 | 40.1±1.5 | 9.1±2.5 | 43.4±2.7 | 41.6±1.6 |
| **II** | **FBP** | -1003.2±5.9 | 30.3±2.2 | 43.0±3.8 | 31.3±3.8 | 37.3±1.5 | 6.6±2.0 | 45.4±3.7 | 40.8±3.2 |
|  | **FBP+PS** | -1002.1±5.8 | 30.4±2.2 | 42.9±3.8 | 31.1±3.8 | 37.2±1.5 | 6.7±1.9 | 45.4±3.7 | 40.4±3.1 |
|  | **I3** | -1003.0±5.9 | 30.4±2.2 | 42.9±3.8 | 31.4±3.8 | 37.3±1.4 | 6.4±1.9 | 45.2±3.7 | 40.8±3.2 |
|  | **I3+PS** | -1001.9±5.9 | 30.4±2.2 | 42.9±3.8 | 31.1±3.8 | 37.3±1.5 | 6.5±1.9 | 45.3±3.6 | 40.5±3.1 |
| **III** | **FBP** | -999.9±1.6 | 30.0±2.8 | 42.2±3.7 | 30.5±3.2 | 37.3±2.5 | 6.4±1.8 | 39.8±3.6 | 41.3±3.9 |
|  | **FBP+PS** | -999.0±1.6 | 30.0±2.9 | 42.2±3.7 | 30.3±3.3 | 37.2±2.5 | 6.6±1.7 | 39.9±3.6 | 41.2±4.0 |

**Table S2.** Relative difference in % between mean HU values for different reconstruction methods.

| **Site** | **Method** | **A** | **FL** | **CG** | **CP** | **NL** | **LV** | **TL** | **VC** |
| --- | --- | --- | --- | --- | --- | --- | --- | --- | --- |
| **I** | **FBP vs. FBP+PS** | -0.1 | 0.1 | 0.1 | -0.3 | -0.5 | 1.5 | 0.1 | -0.5 |
|  | **FBP+PS vs. I3** | 0.1 | 0.0 | 0.7 | 0.8 | 1.1 | -3.9 | 0.6 | 1.6 |
|  | **I3+PS vs. I3** | 0.1 | -0.1 | -0.2 | 0.4 | 0.3 | -0.2 | -0.2 | 0.3 |
| **II** | **FBP vs. FBP+PS** | -0.1 | 0.2 | -0.1 | -0.7 | -0.3 | 2.7 | 0.1 | -1.0 |
|  | **FBP+PS vs. I3** | 0.1 | -0.1 | -0.1 | 0.9 | 0.3 | -5.5 | -0.5 | 1.0 |
|  | **I3+PS vs. I3** | 0.1 | -0.1 | 0.0 | 0.8 | 0.2 | -2.3 | -0.2 | 0.7 |
| **III** | **FBP vs. FBP+PS** | -0.1 | 0.0 | -0.1 | -0.6 | -0.5 | 2.9 | 0.1 | -0.4 |

#####

##### Noise

**Table S3.** Noise (measured by standard deviation of HU values) and their standard deviations for each ROI.

| **Site** | **Method** | **A** | **FL** | **CG** | **CP** | **NL** | **LV** | **TL** | **VC** |
| --- | --- | --- | --- | --- | --- | --- | --- | --- | --- |
| **I** | **FBP** | 2.4 +/- 0.5 | 4.7 +/- 0.7 | 4.4 +/- 0.7 | 4.8 +/- 0.6 | 4.8 +/- 0.6 | 5.0 +/- 1.6 | 4.9 +/- 0.9 | 5.5 +/- 0.8 |
|  | **FBP+PS** | 1.3 +/- 0.3 | 3.9 +/- 0.6 | 3.7 +/- 0.6 | 3.9 +/- 0.6 | 3.9 +/- 0.6 | 4.4 +/- 1.7 | 4.3 +/- 0.7 | 4.7 +/- 0.9 |
|  | **I3** | 2.0 +/- 0.4 | 4.0 +/- 0.7 | 3.7 +/- 0.7 | 4.0 +/- 0.6 | 3.9 +/- 0.6 | 4.3 +/- 2.0 | 4.4 +/- 0.8 | 4.8 +/- 0.9 |
|  | **I3+PS** | 1.4 +/- 0.3 | 3.8 +/- 0.7 | 3.7 +/- 0.6 | 3.9 +/- 0.6 | 3.8 +/- 0.6 | 4.3 +/- 2.0 | 4.4 +/- 0.7 | 4.6 +/- 0.9 |
| **II** | **FBP** | 2.8 +/- 1.0 | 4.0 +/- 0.6 | 4.8 +/- 0.8 | 4.5 +/- 0.6 | 4.1 +/- 0.5 | 4.4 +/- 1.1 | 5.9 +/- 1.0 | 5.3 +/- 0.9 |
|  | **FBP+PS** | 2.2 +/- 0.9 | 3.8 +/- 0.6 | 4.2 +/- 0.8 | 4.0 +/- 0.6 | 3.8 +/- 0.4 | 4.1 +/- 1.2 | 5.7 +/- 1.0 | 4.7 +/- 0.8 |
|  | **I3** | 2.4 +/- 1.1 | 3.2 +/- 0.6 | 4.1 +/- 0.9 | 3.6 +/- 0.6 | 3.1 +/- 0.4 | 3.5 +/- 1.3 | 5.3 +/- 1.1 | 4.4 +/- 1.0 |
|  | **I3+PS** | 2.4 +/- 1.0 | 3.2 +/- 0.6 | 4.1 +/- 0.9 | 3.6 +/- 0.6 | 3.1 +/- 0.4 | 3.5 +/- 1.3 | 5.3 +/- 1.1 | 4.4 +/- 0.9 |
| **III** | **FBP** | 2.5 +/- 1.1 | 4.0 +/- 0.6 | 4.9 +/- 0.9 | 4.9 +/- 0.8 | 4.1 +/- 0.6 | 4.2 +/- 1.0 | 4.5 +/- 0.8 | 5.2 +/- 1.1 |
|  | **FBP+PS** | 1.9 +/- 1.0 | 3.6 +/- 0.5 | 4.3 +/- 0.9 | 4.3 +/- 0.8 | 3.6 +/- 0.5 | 3.8 +/- 1.1 | 4.2 +/- 0.9 | 4.5 +/- 1.1 |

**Table S4.** Relative difference in % between noise (measured by standard deviations of HU values) for different reconstruction methods.

| **Site** | **Method** | **A** | **FL** | **CG** | **CP** | **NL** | **LV** | **TL** | **VC** |
| --- | --- | --- | --- | --- | --- | --- | --- | --- | --- |
| **I** | **FBP vs. FBP+PS** | -93.3 | -20.8 | -22.0 | -23.1 | -21.8 | -16.7 | -14.1 | -18.7 |
|  | **FBP+PS vs. I3** | 36.5 | 0.3 | 1.4 | 1.1 | -0.4 | -3.6 | 0.9 | 1.0 |
|  | **I3+PS vs. I3** | 28.5 | 3.4 | 1.8 | 2.9 | 2.9 | 0.7 | 0.5 | 2.7 |
| **II** | **FBP vs. FBP+PS** | -28.2 | -7.1 | -13.5 | -14.7 | -8.5 | -8.6 | -3.6 | -13.7 |
|  | **FBP+PS vs. I3** | 3.7 | -20.9 | -5.7 | -11.2 | -23.0 | -19.3 | -8.5 | -6.8 |
|  | **I3+PS vs. I3** | -5.9 | 0.0 | -1.6 | -1.8 | 0.7 | -0.3 | 0.0 | -1.3 |
| **III** | **FBP vs. FBP+PS** | -42.4 | -12.5 | -14.6 | -16.1 | -12.7 | -11.6 | -7.2 | -17.1 |

#####

##### Signal-to-noise ratio

**Table S5.** Signal-to-noise ratio and their standard deviations for each ROI.

| **Site** | **Method** | **A** | **FL** | **CG** | **CP** | **NL** | **LV** | **TL** | **VC** |
| --- | --- | --- | --- | --- | --- | --- | --- | --- | --- |
| **I** | **FBP** | -438.0 +/- 75.8 | 6.7 +/- 1.0 | 10.1 +/- 1.4 | 6.6 +/- 0.7 | 8.5 +/- 1.2 | 1.9 +/- 0.5 | 8.9 +/- 1.3 | 7.6 +/- 1.1 |
|  | **FBP+PS** | -834.2 +/- 220.4 | 8.1 +/- 1.5 | 12.2 +/- 2.0 | 8.1 +/- 1.1 | 10.3 +/- 1.7 | 2.3 +/- 0.5 | 10.1 +/- 1.5 | 9.0 +/- 1.7 |
|  | **I3** | -509.1 +/- 86.8 | 8.1 +/- 1.5 | 12.1 +/- 1.9 | 8.0 +/- 1.0 | 10.4 +/- 1.6 | 2.3 +/- 0.7 | 10.1 +/- 1.6 | 9.0 +/- 1.5 |
|  | **I3+PS** | -730.7 +/- 178.3 | 8.4 +/- 1.5 | 12.4 +/- 2.0 | 8.2 +/- 1.2 | 10.7 +/- 1.8 | 2.3 +/- 0.6 | 10.2 +/- 1.5 | 9.3 +/- 1.8 |
| **II** | **FBP** | -402.4 +/- 123.0 | 7.7 +/- 1.3 | 9.2 +/- 1.6 | 7.0 +/- 1.2 | 9.3 +/- 1.2 | 1.5 +/- 0.4 | 7.9 +/- 1.1 | 8.0 +/- 1.4 |
|  | **FBP+PS** | -517.4 +/- 195.1 | 8.3 +/- 1.5 | 10.4 +/- 1.7 | 8.0 +/- 1.5 | 10.0 +/- 1.3 | 1.7 +/- 0.5 | 8.2 +/- 1.2 | 9.0 +/- 1.8 |
|  | **I3** | -485.2 +/- 163.0 | 10.0 +/- 2.0 | 11.0 +/- 2.3 | 9.0 +/- 1.8 | 12.4 +/- 1.7 | 1.9 +/- 0.6 | 8.8 +/- 1.5 | 9.7 +/- 2.1 |
|  | **I3+PS** | -467.2 +/- 157.4 | 10.0 +/- 2.0 | 10.8 +/- 2.2 | 8.8 +/- 1.7 | 12.4 +/- 1.8 | 2.0 +/- 0.6 | 8.9 +/- 1.5 | 9.5 +/- 2.0 |
| **III** | **FBP** | -439.1 +/- 110.7 | 7.7 +/- 1.3 | 9.0 +/- 1.8 | 6.4 +/- 1.1 | 9.3 +/- 1.4 | 1.6 +/- 0.5 | 9.2 +/- 1.6 | 8.3 +/- 2.0 |
|  | **FBP+PS** | -612.3 +/- 198.5 | 8.6 +/- 1.5 | 10.3 +/- 2.4 | 7.4 +/- 1.6 | 10.4 +/- 1.6 | 1.8 +/- 0.6 | 9.8 +/- 1.8 | 9.7 +/- 2.6 |

**Table S6.** Relative difference in % between signal-to-noise ratios for different reconstruction methods.

| **Site** | **Method** | **A** | **FL** | **CG** | **CP** | **NL** | **LV** | **TL** | **VC** |
| --- | --- | --- | --- | --- | --- | --- | --- | --- | --- |
| **I** | **FBP vs. FBP+PS** | 45.2 | 16.4 | 17.0 | 17.5 | 16.4 | 14.6 | 11.9 | 14.3 |
|  | **FBP+PS vs. I3** | -64.8 | -1.2 | -1.7 | -1.2 | 0.6 | -1.4 | -0.9 | -0.0 |
|  | **I3+PS vs. I3** | 28.5 | 3.4 | 1.8 | 2.9 | 2.9 | 0.7 | 0.5 | 2.7 |
| **II** | **FBP vs. FBP+PS** | 18.9 | 6.4 | 11.2 | 11.7 | 7.2 | 9.7 | 3.5 | 10.7 |
|  | **FBP+PS vs. I3** | -8.1 | 16.7 | 4.4 | 10.1 | 18.3 | 10.8 | 7.1 | 6.7 |
|  | **I3+PS vs. I3** | 1.2 | -0.1 | 1.5 | 2.2 | -0.7 | -2.4 | -0.3 | 1.8 |
| **III** | **FBP vs. FBP+PS** | 23.3 | 10.3 | 11.7 | 12.3 | 9.9 | 11.7 | 6.1 | 13.2 |
